# 1. Sarcoptic Scabies of the Horse. 2. Psoroptic Scabies of Cattle in Montana1Read at the Thirty-seventh Annual Meeting of the A. V. M. A., Detroit, Michigan, September, 1900.

**Published:** 1900-10

**Authors:** M. E. Knowles

**Affiliations:** Helena, Mont.


### THE JOURNAL

OF

### COMPARATIVE MEDICINE AND VETERINARY ARCHIVES.

VOL. XXI. *OCTOBER, 1900.* No. 10.

### 1. SARCOPTIC SCABIES OF THE HORSE. 2. PSOROPTIC SCABIES OF CATTLE IN MONTANA.[1]

*Sarcoptes scabiei*, Var. Equi. *Psoroptes communis*, Var. Bovis.

BY M. E. KNOWLES, D.V.S.,
HELENA, MONT.

1. SARCOPTIC SCABIES OF THE HORSE. During the early summer of 1896 my attention was called to the existence of a skin disease among horses in Teton County, Montana, supposed to be contagious by the gentleman making the report. A few days after the notice was received I visited Teton County, where a number of the affected animals were examined and were found to be suffering with sarcoptic scabies. On careful inquiry among a number of owners, I ascertained that the disease had been in existence throughout that county since 1884 or 1885; in any event it was about those years when it was first noticed and given attention by one or two of the range horse-owners of the county. It seems that the attention of my predecessors had never been called to the existence of the disease; at any rate there are no records in the office making mention of the disease as having received official attention. As the disease was found to be prevalent throughout the county among range horses, official notice was immediately given it, the county placed in quarantine, and preparations rapidly made toward stamping it out. A number of deputy inspectors (laymen) were appointed by me in various localities in the county, and after a meeting with the owners at Choteau, the county seat, during June of this year, an agreement was reached by which the work progressed rapidly and satisfactorily.

[1] Read at the Thirty-seventh Annual Meeting of the A. V. M. A., Detroit, Michigan, September, 1900.

I was unable to obtain a satisfactory history of the introduction of the disease, but it is assumed that it was introduced from Oregon or Washington through Indian horses; possibly by the Nes Perces Indians during their raid through Montana in 1877, as a number of the bucks of this band of Indians, after General Miles' battle with them on the Snake Creek, drifted over into the Blackfeet Reservation, in the northern portion of Teton County. The existence of the disease under range conditions is mainly interesting from the fact that it is unusual under such conditions, and that it seems to be confined to that territory in Teton County, which, in fact, comprises the major portion of the county, between Sun River on the south and the Marias on the north. At least I have never been able to discover it outside of these boundaries, or, in fact, outside of Teton County.

During the past three years about 12,000 horses have been treated, and the disease is now practically stamped out. The method of handling these horses was as follows: During the spring and fall roundups, each roundup outfit would rope and throw every diseased horse coming under its observation, when, after being secured, they were hand-treated. In fact, every horse treated was unbroken, with one or two exceptions. We were compelled to treat them in this manner because no dipping-vats were constructed. Horses being of so little value at the time my attention was first called to the disease, the owners did not feel justified in building expensive dipping-plants for the purpose of treating the disease, as I suggested they do; but thought that hand-treating would be more economical and quite as efficient. There is no doubt that had vats been constructed the disease could have been more promptly stamped out.

The symptoms of sarcoptic scabies are manifest at first by an intense pruritus. The animal is found rubbing himself by every available means. On examination of the skin the observer will note papules at all the pruriginous points. On passing the hand over the parts, minute elevations will be felt, which consist of a small crust around several hairs. When this is removed there will be found a moist, denuded, red surface. In points between the crusted patches will be found slightly salient papules on close observation, which is the first evidence of the before-mentioned lesion; the papule elevating the epidermis by serous effusion which slowly dries, forming the crust. These lesions disseminate and bring about a loss of hair in small areas, which extend, ultimately becoming confluent, forming large, dry areas covered with epidermic débris and thin crusts. Vesicles rarely appear, as the irritation produced by the pruritus rapidly replaces them by the formation of crusts. By rapid generalization the depilated patches extend, finally invading almost the entire surface of the body, with the exception of the region below the hock and knees. When the disease has made this progress the animal becomes a miserable-looking object; the coat is dead, and over large areas ulcers of various sizes are formed from the constant rubbing, and the irritated surface constantly bleeds. The skin, as would be surmised, is thickened, and, in the region of the neck and other localities where it is attached by loose connective tissue, appears in thick folds. The extensive irritation from constant rubbing and scraping is an important factor in increasing these extensive lesions in the skin.

The parasites in all stages of development are found among the crusts on all the invaded areas. The ovigerous females and the young larvæ live in the epidermic galleries, or tunnels, which are not easily distinguished in any but white-skinned horses. Neumann says that these galleries, or tunnels, are excavated immediately after copulation, and that they are rapidly formed; in from fifteen to thirty minutes the work is completed, and all the more promptly so if the temperature be elevated. He says it is for this reason that the parasites have been considered as noctambulant. If they torment animals more by night than by day, it is because of the condition of the temperature—the warm stable, litter, and probably clothing. As the stable, litter, and clothing do not act as factors under the conditions that I am describing, the above statement does not seem to be true, as horses on a range under constant night and day herd, seem to show about equal irritation at all times. During cold or windy weather it is extremely difficult to discover the sarcoptes; therefore, to not meet with disappointment, it is advisable to have warm, sunshiny weather in which to make the search. It is necessary to make skin-scrapings to the blood and through the entire thickness of the epidermis with a sharp instrument. When the scrapings have been collected they should be placed in a well-heated box or in a window exposed to a strong sunlight. A small quantity of the débris, after it is well warmed, can be spread on a slide and examined with a microscope with one-third or two-thirds objective. Should the slide and stage be warm the sarcoptes will disengage themselves from the débris, when they may be removed with a needle and placed with a drop of glycerin on another glass. Sarcoptic scabies is not as rapid in its development as is psoroptic scabies, and the disease is more clearly shown five or six weeks after its inception.

An interesting fact in connection with this outbreak was two cases of undoubted transmission of the sarcoptic scabies of horse to man. Two horse-wranglers employed by the "F" outfit contracted the disease from riding mangy horses. Both received the infection primarily in the genital region, the disease rapidly extending to the groin and the upper and inner aspect of the thighs, the lower portion of the abdomen, pubes, scrotum, and in one case the penis became involved. Both of these cases were easily cured by the remedy employed in treating the horse.

2. Psoroptic Scabies of the Ox. During May, 1898, it was reported to me by Deputy State Veterinarian, Dr. Thomas Brooks, of Choteau, Teton County, Montana, that there was in existence on the ranges of that county among cattle some apparently contagious skin-disease. On investigating the report I found that the doctor was correct, and that we had psoroptic scabies to deal with under range conditions and over a very wide area. Investigating the history of this disease on the northern Montana ranges, I ascertained from reputable cattlemen who had been in the range-cattle business for years, that the disease had existed to some extent for at least twenty years, and was generally regarded by experienced cattlemen as of little or no consequence, but it was admitted that it had been increasing during the past two or three years. At about the same time I ascertained from cattlemen ranging their cattle across the line in Alberta, N. W. T., that the disease was quite as prevalent in that locality, the approximate boundaries in which the disease exists being from Sun River, in Montana, as far north as the Saskatchewan in Alberta, and from the Rocky Mountains well toward the Dakota line on the east. Our range cattle-owners have been led to attach little importance to it, for the reason that the disease shows itself plainly during cold weather only, many cases having a tendency to apparently recover during the warmer months of the year of their own accord, and to this alone is probably responsible the fact that the disease was not reported and investigated long ago.

The Canadian authorities in Alberta, I learn from Dr. Thomas Wroughton and Dr. John Burnett, veterinarians and lieutenants in the Northwest Mounted Police, made the discovery of the existence of this disease at about the time it was reported to me by Dr. Brooks. They immediately compelled the cattle-owners of Alberta to construct three dipping-vats after the plans recommended by the United States Bureau of Animal Industry, and commenced dipping in Zundell's dip, which I believe did not prove so satisfactory as was anticipated or as they desired.

On account of the difficulty in convincing our cattle-owners of the necessity for the above-mentioned procedure, no vats were constructed in this State until this year, and up to date we have but two in operation. During the past two years a great many cattle have been treated throughout the described area by what is commonly known among sheep-men as hand-patching, and in the main the method has proved successful, although expensive and tedious.

Neumann says that Linnæus observed on cattle a mange that was caused and maintained by insects which were found located in the tubercles of the skin; but in reality the first mention of the parasite (1813) is due to Dorfuille, a French veterinarian, Port St. Marie, who reported his discovery to Gohier, who in the following year found this acarus on Hungarian cattle which the Austrian army brought in large numbers to Lyons. Gohier regarded this parasite as identical with *Psroptes communis*, Var. Equi., with which he was familiar. Delafond, in 1866, discovered this parasite on English and Limousin cattle at about the same time that Gerlach discovered it on mangy cattle at Bomberg. Muller discovered it in 1860, and at intervals since that time it has been discovered in France. Fleming says the disease is not common in the United Kingdom. Dr. Mayo mentions its existence in Kansas, stating that he suspected its introduction from Texas cattle, and the disease there he says is commonly known as "Texas itch."

I am informed by reputable cattlemen that it exists in Wyoming, and widely, also, in Montana and the Northwest Territory as I have already stated. A number of our reputable cattlemen who have been in the range-cattle business for thirty years or more believe that the infection came from the buffalo, and described the disease as having affected buffaloes in the same manner. Old buffalo-hunters still residing in Montana have described to me such a skin affection, claiming to have killed buffaloes where the hide was worthless from a complete absence of hair, and covered with ulcers. They also relate that the disease appeared to be worse on the neck, withers, and the base of the tail. While it is quite possible that buffaloes suffered from some form of scabies, and that they have been responsible for the contagion, it seems strange that it should be perpetuated among range cattle in the area mentioned, when the buffaloes roamed over the entire western plains from Texas practically to the Arctic circle, and from the Rocky Mountains to the Missouri River. Roll, a Continental veterinarian, claims to have seen the disease on buffaloes, doubtless having reference to the European buffalo. We have yet remaining in Montana 500 to 600 head of buffalo under close domestication which I have examined, and have never found the slightest evidence of any skin disease of a contagious character existing among them.

The disease among cattle makes its first appearance at the root of the tail, or on the neck and withers, extending to the shoulders, neck, sides of chest, and in long-standing cases to the entire body and head, except the extremities below the knee and hock, in which regions I have never observed it. On the invaded areas small elevations of the skin are observed, single or running together, from which exudes serum, which dries and forms in successive layers of hard crusts, under and between which the parasites are found. The skin is thickened, hard and dry in localities, and on the neck, withers and sides of chest thick folds will be observed. The animal rubs and scratches by every available means. The pruritus is intense. I have observed animals scratching on wire fences, frequently cutting the skin through for a distance of from 8 to 14 inches on the sharp barbs. They are also observed scratching and rubbing against each other, in this manner certainly spreading the contagion. When an animal is in an advanced stage of the disease it takes réfuge near an object on which it can rub, and will not leave that object for sufficient time to graze and obtain necessary sustenance. In advanced cases the animal is almost totally devoid of hair, death occurring in the winter from freezing, the animal being anæmic and debilitated. The disease is markedly prevalent among range bulls, from the fact that they come in contact with a greater number of cattle than the other sex.

Neumann states that Gerlach and Muller remarked on the modifications occurring in this form of scabies by change of season, reciting an instance where the disease prevailed among cattle every year, commencing toward the end of autumn, when the animals began to be housed, reaching its maximum in February, and diminishing in the spring, when the oxen were employed at labor; the crusts then became detached, the hairs grew, and there then only remained a few patches at the root of the tail and on the neck. The train of symptoms reappeared each autumn. The same writer also observes that these parasites (psoroptes) persist during the summer, although the cattle appeared to be cured. They (Gerlach and Muller), found the parasite in great numbers on the neck and around the horns. I have found great numbers of the parasite on cattle during the summer months, and was surprised at the slight amount of skin irritation at this time; in fact, one must observe closely to discover it; but I have observed the disease persisting throughout the summer, and apparent to the most careless observer. Neumann concludes from these facts that the residence of cattle in warm stables during the winter favors the development of the parasite. I cannot agree with the last statement. All the cattle in Montana suffering from this disease are range cattle that never knew a stable from birth to slaughter, and while it is true that the disease in many instances apparently gets well of its own accord during the summer, the parasite still remains, and on the approach of cold weather the disease is aggravated rapidly, due to the fact that the acarus requires more food during cold weather, making it, perforce, more active, and there is no doubt the secretions of the parasite are more irritating in cold than during warm weather.

All cattlemen of long experience with this disease on Montana ranges maintained, until shown the psoroptes, that the disease was due to hard "winters;" from the freezing and thawing of snow accumulating on the backs of the cattle, and long-continued, cold winters. They say the disease has been particularly noticeable after our very hard winters, and that after an open, mild winter but few cases of it have been observed. It is also said the disease is much worse on the open plains than where there is shelter, such as is afforded by the breaks along the Missouri, Marias, Teton, and other streams, and in the mountains. My observation has been that the disease persists with equal severity on the plains and in the sheltered places.

It is difficult, under range conditions, to arrive at an accurate estimate of the number affected with the disease, but, from my observations during the past year, would regard as a conservative estimate of cattle ranging north of the Missouri River, and from the mountains to the Dakota line, that about 20 per cent. are affected. This estimate refers to cattle plainly showing the disease, but there is no doubt that equally as great, or greater, percentages are affected that during the warm summer months show no evidence of it until cast and carefully examined.

Paraffin oil, to which was added about 3 per cent. of sulphur where hand-treating was employed, has proved to be a very efficient remedy for both psoroptic and sarcoptic scabies. Since the dipping-vats have been constructed a lime-and-sulphur dip is employed cheaply and successfully.

